# Pheochromocytoma-induced myocardial infarction: A case report

**DOI:** 10.1515/biol-2022-0830

**Published:** 2024-02-24

**Authors:** Haixia Tang, Jichun Liu, Bangsheng Hu, Yuwen Yang, Xiangrong Xie, Youquan Wei

**Affiliations:** Department of Cardiology, The First Affiliated Hospital of Wannan Medical College, Wuhu 241000, Anhui, China

**Keywords:** pheochromocytoma, acute myocardial infarction, chest pain, case report, neuroendocrine tumor

## Abstract

The pheochromocytoma is an uncommon endocrine neoplasm that originates from chromaffin cells and causes significant cardiovascular effects through the intermittent or sustained release of catecholamines. In this report, we present a rare case of myocardial infarction (MI) induced by pheochromocytoma. A 53-year-old female presented to the emergency department with a history of intermittent palpitations, back pain, and sweating for over 10 years, which had worsened over the past 2 days. The patient’s cardiac enzymes and troponin levels were significantly elevated, and the electrocardiogram (ECG) showed ST-segment elevation, leading to an initial diagnosis of acute myocardial infarction. Echocardiography revealed apical ballooning, indicative of stress cardiomyopathy. Emergency coronary angiography revealed no significant stenosis, and the patient’s blood pressure was fluctuating. Computerized tomography (CT) scan of the adrenal gland revealed a bilateral adrenal mass, with the left adrenal mass being larger in size after contrast-enhanced CT scan. The patient’s left adrenal gland was successfully removed through laparoscopic adrenalectomy, and histopathology results confirmed the presence of adrenal pheochromocytoma. Follow-up for 3 months after discharge showed the patient had no symptoms and good prognosis. The abnormal findings on echocardiography and ECG resolved. Prompt diagnosis and management of pheochromocytoma are crucial for a favorable prognosis.

## 1 Background

Pheochromocytoma is a rare neuroendocrine tumor originating from the adrenal medulla, known for its tendency to cause abnormal catecholamine secretion and resulting in various cardiovascular complications [1]. These tumors exert significant cardiovascular effects by intermittently or continuously releasing catecholamines. The clinical manifestations of pheochromocytoma are varied, and the classic triad of paroxysmal headache, palpitations, and sweating is only present in 24% of cases, leading to misdiagnosis by clinicians [2]. In this report, we present a rare case of myocardial infarction (MI) induced by pheochromocytoma. The objective of our research is to investigate the clinical characteristics, diagnostic complexities, and therapeutic strategies associated with this case, thereby enhancing comprehension and providing a valuable reference for the efficient management of this condition.

## 2 Case presentation

**Figure 1:**
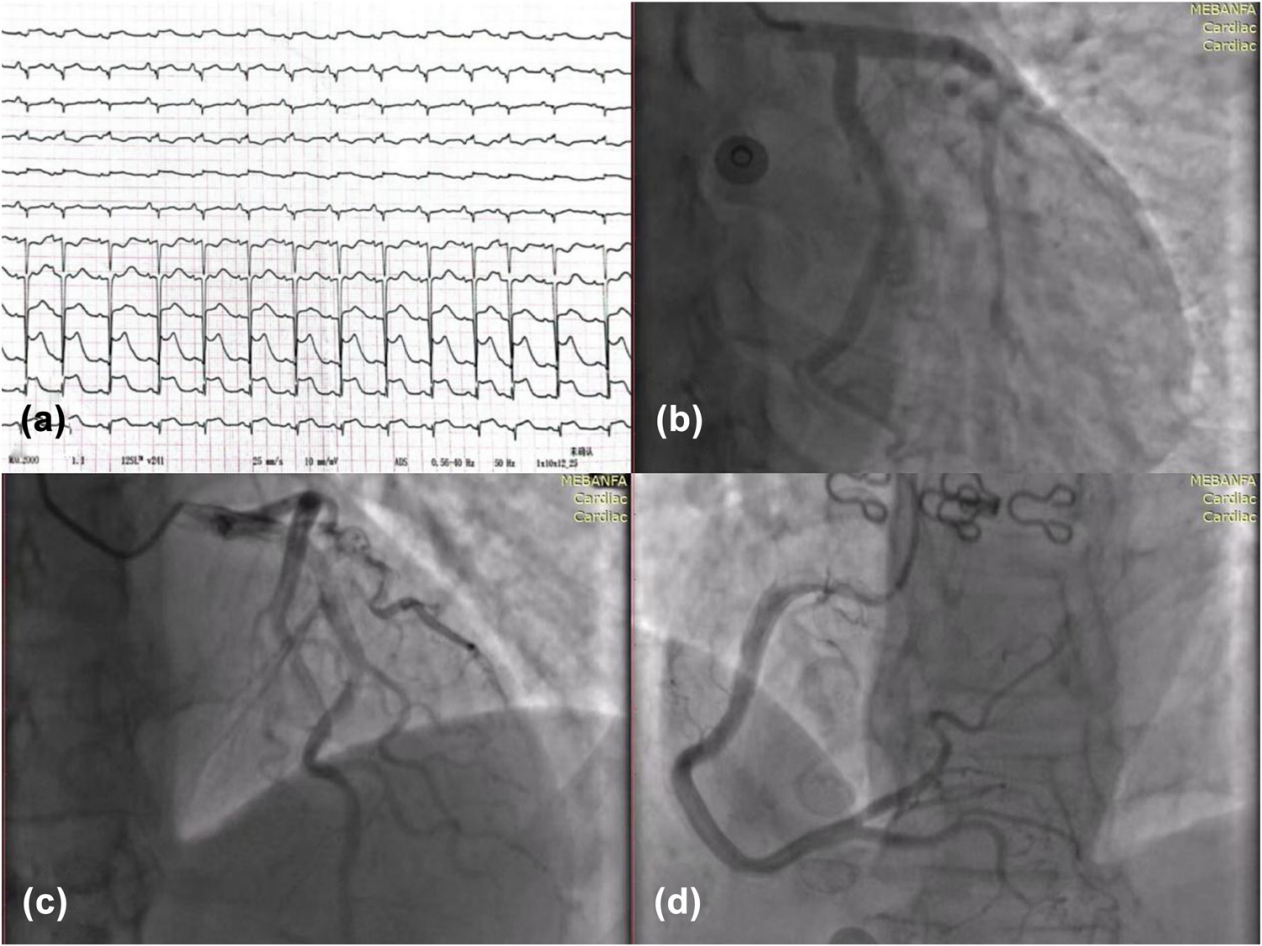
Results of the ECG and coronary angiography. The ECG showed normal sinus rhythm and ST-segment elevations in leads II, III, aVF, and V2-V6 (a). A coronary angiography revealed no coronary stenosis (b–d).

A 53-year-old woman with a history of palpitation, back pain, and sweating for over 10 years presented to the hospital with worsening symptoms for the past 2 days. The patient had no preexisting history of hypertension or diabetes, but did have a history of radiofrequency ablation for atrial fibrillation. Upon admission, her vital signs showed a body temperature of 36.5°C, respiratory rate of 19/min, pulse rate of 78/min, blood pressure of 200/140 mmHg, and oxygen saturation of 99%. Her creatine kinase isoenzyme was 144 U/L (normal range: 0–25 U/L) and troponin I was 12.412 ng/mL (normal range: 0–0.03 ng/mL). The electrocardiogram (ECG) revealed sinus rhythm with ST-segment elevation observed in leads II, III, aVF, and V2–V6 (Figure 1a), leading to a preliminary diagnosis of acute myocardial infarction (AMI).

**Figure 2:**
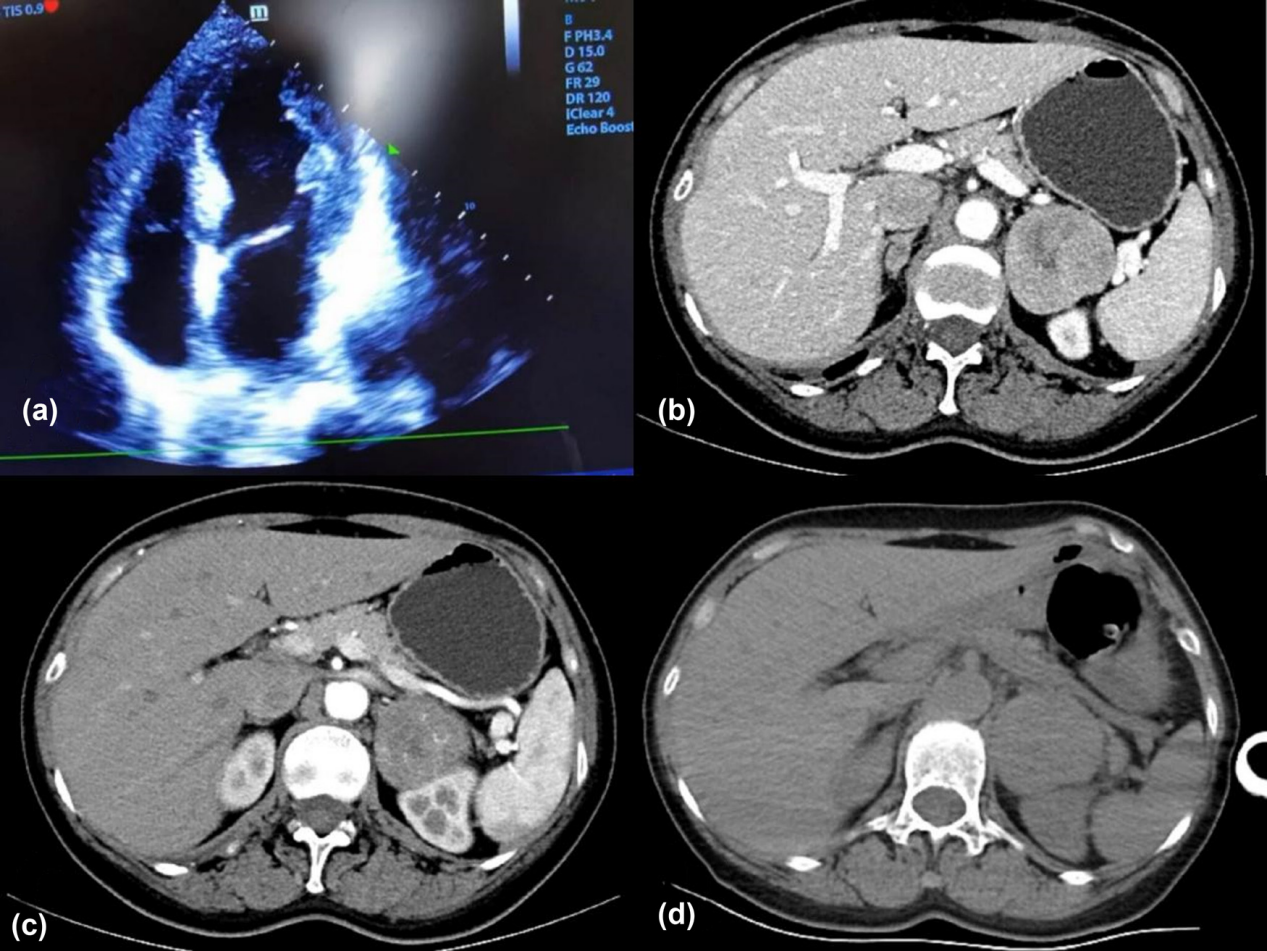
Results of echocardiography and adrenal gland CT scan. Echocardiography showed apical bulbous motion with an EF of 63% (a). CT scan of the adrenal gland revealed bilateral adrenal mass, with the left adrenal mass being larger in size (b–d).

However, emergency coronary angiography revealed no coronary stenosis (Figure 1b–d) and an emergency cardiac ultrasound showed apical bulbous motion with an EF of 63% (Figure 2a), suggesting stress cardiomyopathy. Computerized tomography (CT) scan of the adrenal gland revealed a bilateral adrenal mass (left side size: 52.2 mm × 53.6 mm, right side size: 18.8 mm × 10.9 mm), with the left adrenal mass being larger in size after enhanced CT scan (Figure 2b–d). The patient was given antihypertensive treatment due to her high blood pressure upon admission, and phentolamine was administered orally in the morning due to the suspicion of pheochromocytoma. Regular monitoring of blood pressure, myocardial enzyme profile, troponin, and ECG was conducted. After other subsequent examinations, dopamine levels were improved to 2.80 pg/mL (normal range: <30.00), norepinephrine to 1923.20 pg/mL (normal range: decubitus, 70–750 pg/mL; standing position, 200–1,700 pg/mL), and epinephrine to 3046.20 pg/mL (normal range: decumbent position ≤111 pg/mL, upright position ≤141 pg/mL). Genetic testing indicated that TMEM127 was pathogenic. The results of all the auxiliary tests strongly indicated a diagnosis of pheochromocytoma, and based on the multidisciplinary consultation, it was recommended to perform a laparoscopic left adrenalectomy.

**Figure 3:**
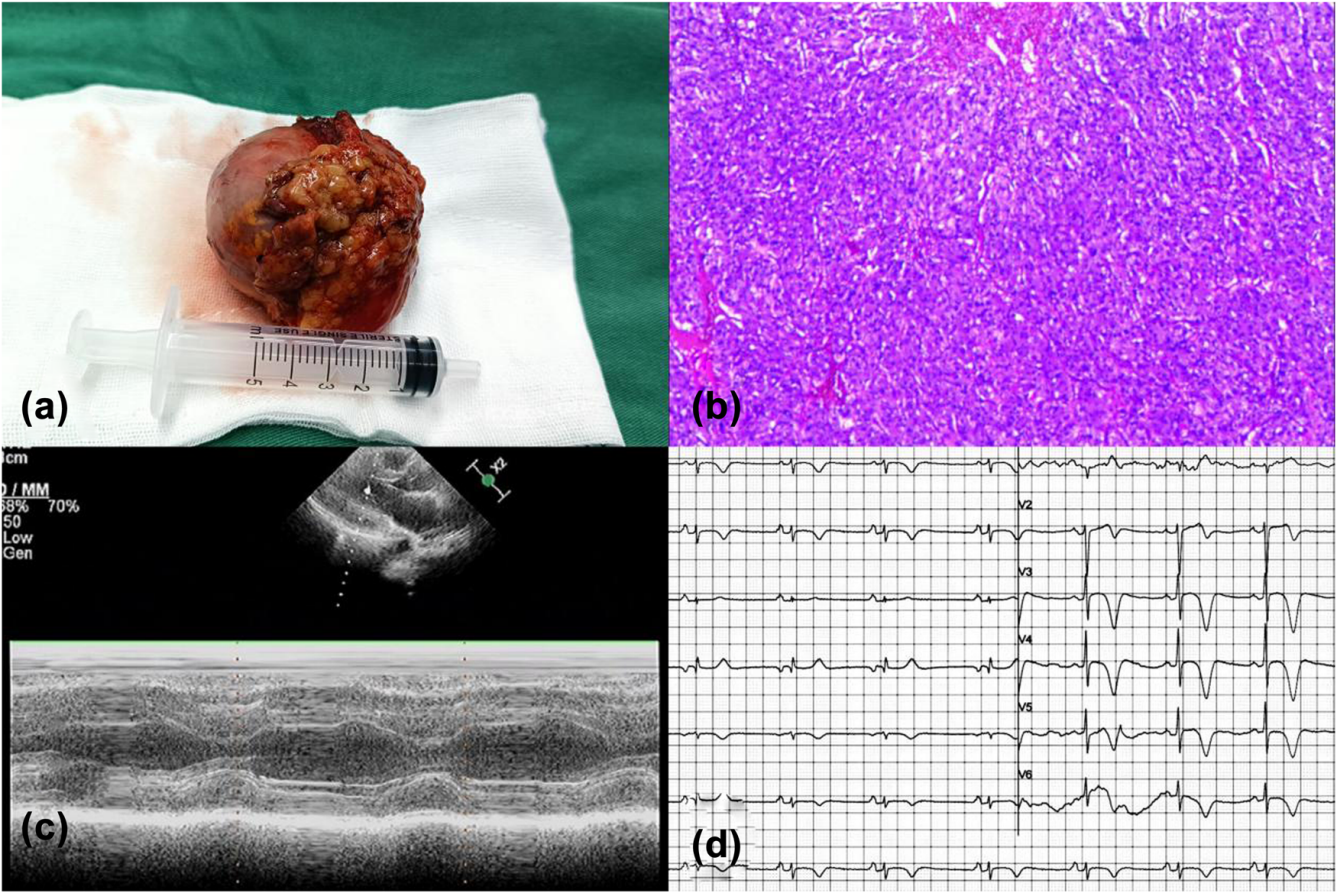
Specimen from left adrenalectomy (a) and histopathological examination confirmed adrenal pheochromocytoma (b). Results of postoperative ECG and echocardiography (c and d).


General anesthesia is administered to ensure the patient remains unconscious and pain-free during the surgery. Small incisions are made in the abdomen, through which a laparoscope and other specialized instruments are inserted. The surgeon uses these instruments to dissect and remove the affected adrenal gland containing the pheochromocytoma. Finally, the patient underwent a successful laparoscopic left adrenalectomy (Figure 3a). Histopathological examination of the specimen confirmed the presence of adrenal pheochromocytoma (Figure 3b). In the postoperative examination, her creatine kinase isoenzyme was 19 U/L, troponin I was 0.056 ng/mL, and the ST of each ECG lead was lower than before (Figure 3c). An echocardiogram indicated weakened apical motion, but the remaining ventricular wall motion was not significantly abnormal at present, with an EF of 59% (Figure 3d). Follow-up for 3 months after discharge showed the patient had no symptoms and good prognosis. The abnormal findings on both echocardiography and ECG resolved.

**Informed consent:** Informed consent has been obtained from all individuals included in this study.
**Ethical approval:** The research related to human use has been complied with all the relevant national regulations, institutional policies and in accordance with the tenets of the Helsinki Declaration, and has been approved by the authors' institutional review board or equivalent committee.

## 3 Discussion

The presented case highlights the importance of considering pheochromocytoma as a differential diagnosis in patients with atypical cardiac symptoms and unexplained hypertension, even in the absence of classic symptoms such as paroxysmal hypertension and headache. Prompt recognition and surgical intervention led to a successful outcome in this patient. Pheochromocytoma-induced MI is a rare clinical entity that poses diagnostic challenges and requires prompt management. In this case report, we discuss the diagnostic considerations and management strategies employed for a patient presenting with pheochromocytoma-induced MI.

The sporadic secretion of catecholamines by pheochromocytoma can cause symptoms such as headache, sweating, palpitations, and elevated blood pressure, with persistent or paroxysmal hypertension being a hallmark feature. Symptoms associated with paroxysmal headache, sweating, and hypertension may be more sensitive and specific for pheochromocytoma than any biochemical test [3–5]. It is now known that at least 30% of these tumors are hereditary, but most cases remain undiagnosed during a patient’s lifetime [6]. The clinical presentation of pheochromocytoma indeed exhibits significant variability, posing a diagnostic challenge when patients do not manifest the classic triad of symptoms. In such cases, clinicians must maintain a high index of suspicion and consider the possibility of this condition. Diagnosis of pheochromocytoma requires a combination of history, clinical presentation, and laboratory findings, especially in the case of atypical symptoms. Therefore, a comprehensive understanding of the different clinical manifestations of this disease is essential for clinicians to effectively identify and manage all manifestations of pheochromocytoma.

Due to the potential life-threatening nature of pheochromocytoma-induced MI, early intervention is necessary to prevent further cardiac damage and complications. Surgical excision is the primary treatment, but given the potential for these tumors to secrete catecholamines, patients are at risk for uncontrolled hypertension, especially during surgery. Therefore, a multidisciplinary approach to preparation and treatment is essential [7]. In addition, preoperative medical management is crucial to stabilize the patient’s hemodynamics and prevent perioperative complications. Alpha-blockers and beta-blockers are commonly used to control blood pressure, suppress catecholamine release, and reduce the risk of intraoperative hypertensive crises [8,9]. The careful monitoring and optimization of the patient’s cardiovascular status are imperative throughout the perioperative period.

ST-segment elevation myocardial infarction (STEMI) is defined as an increase in creatine kinase-myocardial subtypes and troponin *T* values greater than the upper reference limit, typical chest pain lasting >2 min, an ECG showing a new ST-segment elevation of ≥2 mm on at least two consecutive precardiac ECG leads, or a new ST-segment elevation of at least 1 mm on two adjacent limb leads or the presence of a new left bundle branch block [10]. Pheochromocytoma presents with various clinical symptoms and the release of catecholamines can lead to severe cardiovascular complications that have the potential to be life-threatening [11]. The presentation of pheochromocytoma-induced MI can be atypical and mimics other cardiovascular conditions, making its diagnosis challenging. Hypertension is a common symptom in pheochromocytoma, but the presence of MI symptoms, such as chest pain, ECG changes, and elevated cardiac biomarkers, may lead to a diagnostic dilemma [12]. Recognizing pheochromocytoma as a possible underlying cause requires a high level of suspicion, especially in patients with uncontrolled hypertension and non-specific symptoms.

However, clinically diagnosed pheochromocytoma is rare, and presented with AMI is even rarer. Pheochromocytoma-associated cardiomyopathy is a rare disease in which the excessive release of catecholamines, including norepinephrine and epinephrine, from the tumor, can cause severe vasoconstriction, myocardial ischemia, damage, and necrosis. In some cases, it can present as AMI [2,13], as was seen in this patient with symptoms such as palpitations, back pain, sweating, and significantly elevated blood pressure. In our case, the initial diagnosis was acute STEMI, but the coronary angiography showed no acute occlusion of the coronary arteries. Instead, reversible coronary spasm induced by catecholamines was suspected of having caused ischemic and hypoxic myocardial damage.

Diagnosing pheochromocytoma can be difficult due to its varied clinical symptoms. Common cardiovascular manifestations include paroxysmal or persistent hypertension, catecholamine cardiomyopathy, and multiple arrhythmias such as atrial flutter, atrial fibrillation, supraventricular tachycardia, ventricular tachycardia, ventricular fibrillation, and cardiac arrest [2,14]. In our case, the patient did not present with the classical triad of symptoms associated with pheochromocytoma. Instead, the patient experienced an AMI, which resulted in abnormal changes in both the ECG and cardiac structure and function. However, after the surgical removal of the pheochromocytoma, the abnormal ECG and echocardiography findings resolved.

Clinically confirmed cases of pheochromocytoma are rare, and those with AMI are even rarer. Several case reports have reported cardiomyopathy as a complication of pheochromocytoma [2,13,15,16]. The occurrence of MI by pheochromocytoma is rare and clinical reports are limited. When it does occur, electrocardiography may show ST-segment elevation or, more commonly, non-ST-segment elevation MI. In contrast to classical MI, most of these cases exhibit no significant coronary artery atherosclerosis [17]. Pheochromocytoma is a tumor that may have a variety of clinical manifestations and can be secondary to catecholamine effects on different organ systems. However, the concomitant presence of such complications makes the diagnosis and management of this patient particularly challenging [15].

## 4 Conclusion

The findings of this case serve as a valuable reference for the diagnosis and treatment of similar patients in the future. Pheochromocytoma can mimic symptoms seen in various other diseases, highlighting the importance of considering this condition in the differential diagnosis. Early diagnosis and prompt management of pheochromocytoma are crucial, as they can prevent further cardiac damage and complications. This case underscores the significance of timely intervention, which ultimately leads to a more favorable prognosis for affected individuals.
